# Supplementary material for: The effect of giant lateral collapses on magma pathways and the location of volcanism
Source: Nat Commun. 2017 Oct 23;8:1097. doi: 10.1038/s41467-017-01256-2 (PMC5653657; doi:10.1038/s41467-017-01256-2)
Supplement: Supplementary file 1 — Supplementary Information [file 41467_2017_1256_MOESM1_ESM.pdf]

| | ***EVL1*** | | | ***EVL2*** | | | ***EVL3*** | | |
|---|---|---|---|---|---|---|---|---|---|
| | ***Linear*** | ***Quad.*** | ***Cubic*** | ***Linear*** | ***Quad.*** | ***Cubic*** | ***Linear*** | ***Quad.*** | ***Cubic*** |
| ***EVL=0.1*** | 12.5/10 =1.250 | 9.7/10 =0.970 | 8.3/10 =0.830 | 12.5/14 =0.893 | 9.7/14 =0.693 | 8.3/14 =0.593 | 12.5/20 =0.625 | 9.7/20 =0.485 | 8.3/20 =0.415 |
| ***EVL=0.2*** | 12.5/20 =0.625 | 9.7/20 =0.485 | 8.3/20 =0.415 | 12.5/28 =0.446 | 9.7/28 =0.346 | 8.3/28 =0.296 | 12.5/40 =0.313 | 9.7/40 =0.243 | 8.3/40 =0.208 |
| ***EVL=0.4*** | 12.5/40 =0.313 | 9.7/40 =0.243 | 8.3/40 =0.208 | 12.5/56 =0.223 | 9.7/56 =0.173 | 8.3/56 =0.148 | 12.5/80 =0.208 | 9.7/80 =0.162 | 8.3/80 =0.138 |
| ***EVL=0.6*** | 12.5/60 =0.208 | 9.7/60 =0.162 | 8.3/60 =0.138 | 12.5/84 =0.149 | 9.7/84 =0.115 | 8.3/84 =0.099 | 12.5/120 =0.104 | 9.7/120 =0.081 | 8.3/120 =0.069 |

**Supplementary Table 1:** Unloading/loading ratios used in each of the simulations shown in Fig. 5c. The numerators are the cross sectional areas (km$^2$) of the different collapse profiles (linear, quadratic, and cubic). The denominators are the cross sectional areas (km$^2$) of the effective volcanic loading (i.e. elastic loading scaled by EVL magnitude, first column). The corresponding unloading and loading forces (in plane strain condition) are obtained by multiplying the areas by the gravity acceleration and rock density.

| | ***Unloading profile 1*** | ***Unloading profile 2*** | ***Unloading profile 3*** |
|---|---|---|---|
| ***$EVL_1$ = 0.5*** | 12.5/50 ≈ 25% | 9.7/50 ≈ 20% | 8.3/50 ≈ 16% |
| ***$EVL_2$ = 0.6*** | 12.5/84 ≈ 15% | 9.7/84 ≈ 12% | 8.3/84 ≈ 10% |
| ***$EVL_3$ = 0.6*** | 12.5/120 ≈ 10% | 9.7/120 ≈ 8% | 8.3/120 ≈ 7% |

**Supplementary Table 2:** Critical unloading/loading ratios for dyke deflection. As for Supplementary Table 1, the numerators are the cross sectional areas (km$^2$) of the different collapse profiles (1 – linear, 2 – quadratic, and 3 – cubic decay). The denominators are the cross sectional areas (km$^2$) of the effective volcanic loading (i.e. elastic loading scaled by EVL magnitude). The corresponding unloading and loading forces (in plane strain condition) are obtained by multiplying the areas by the gravity acceleration and rock density.

| *Density difference (Δρ).* kg/m$^3$ | *Rock fracture toughness ($K_r$).* MPa x m$^{1/2}$ | *Magma bulk modulus (K).* GPa | *Rock shear modulus (G).* GPa | *Minimum dyke volume to reach the east (E) or west (W) flank of the volcano.* km$^3$ |
|---|---|---|---|---|
| 300 | 100 | 50 | 20 | 2.5 x 10$^{-3}$ (E) |
| **400** | 100 | 50 | 20 | 0.9 x 10$^{-3}$ (E) |
| 300 | **185** | 50 | 20 | 5.5 x 10$^{-3}$ (E) |
| 300 | 100 | **10** | 20 | 4.0 x 10$^{-3}$ (E) |
| 300 | 100 | 50 | **30** | 2.1 x 10$^{-3}$ (E) |
| 300 | 100 | 50 | 20 | 34.3 x 10$^{-3}$ (**W**) |

**Supplementary Table 3:** Minimum volume for a dyke to reach the surface of our model as function of magma and rock parameters.

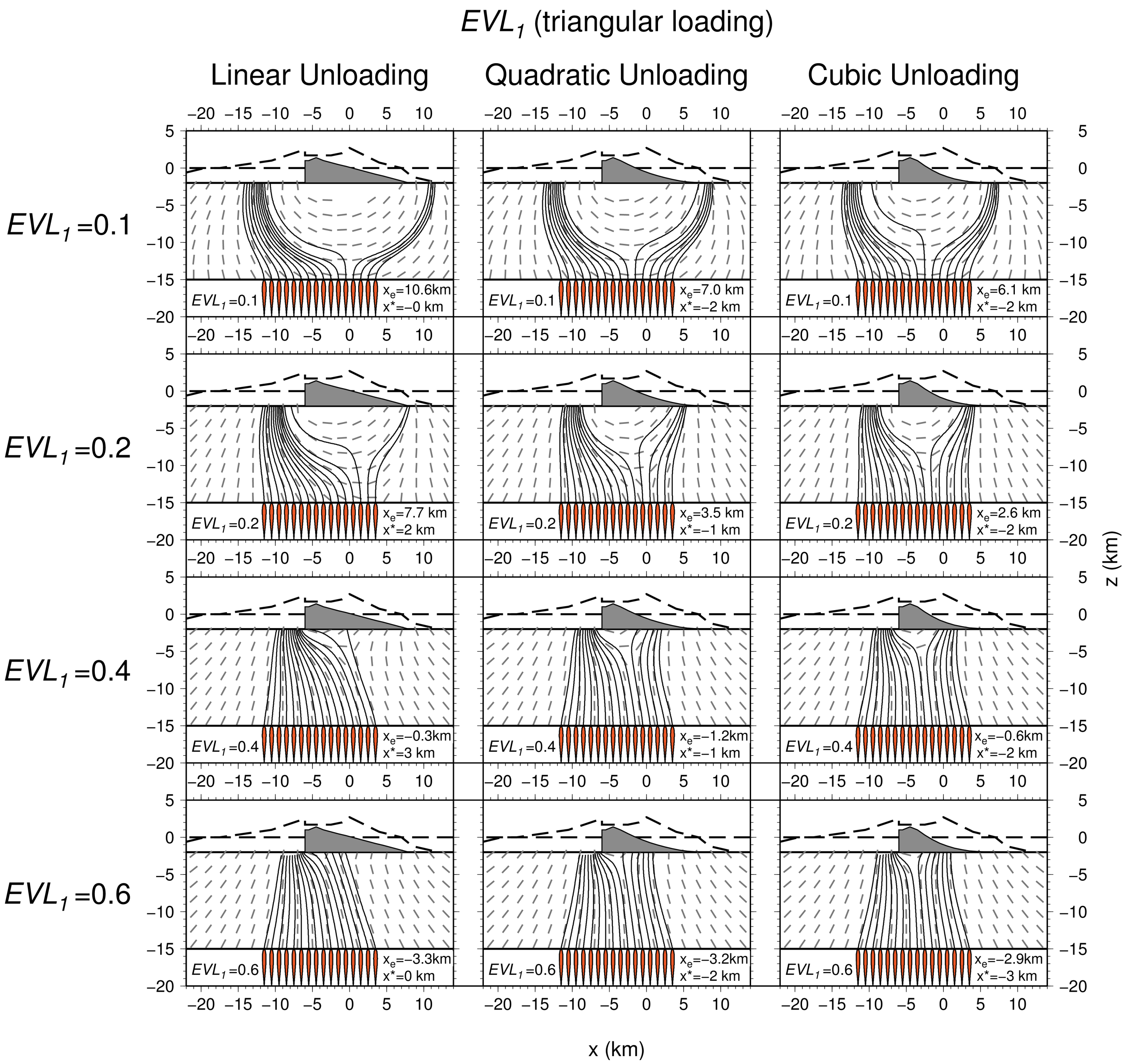


**Supplementary Figure 1:** Model results for $EVL_1$ as function of EVL magnitudes and unloading profiles.

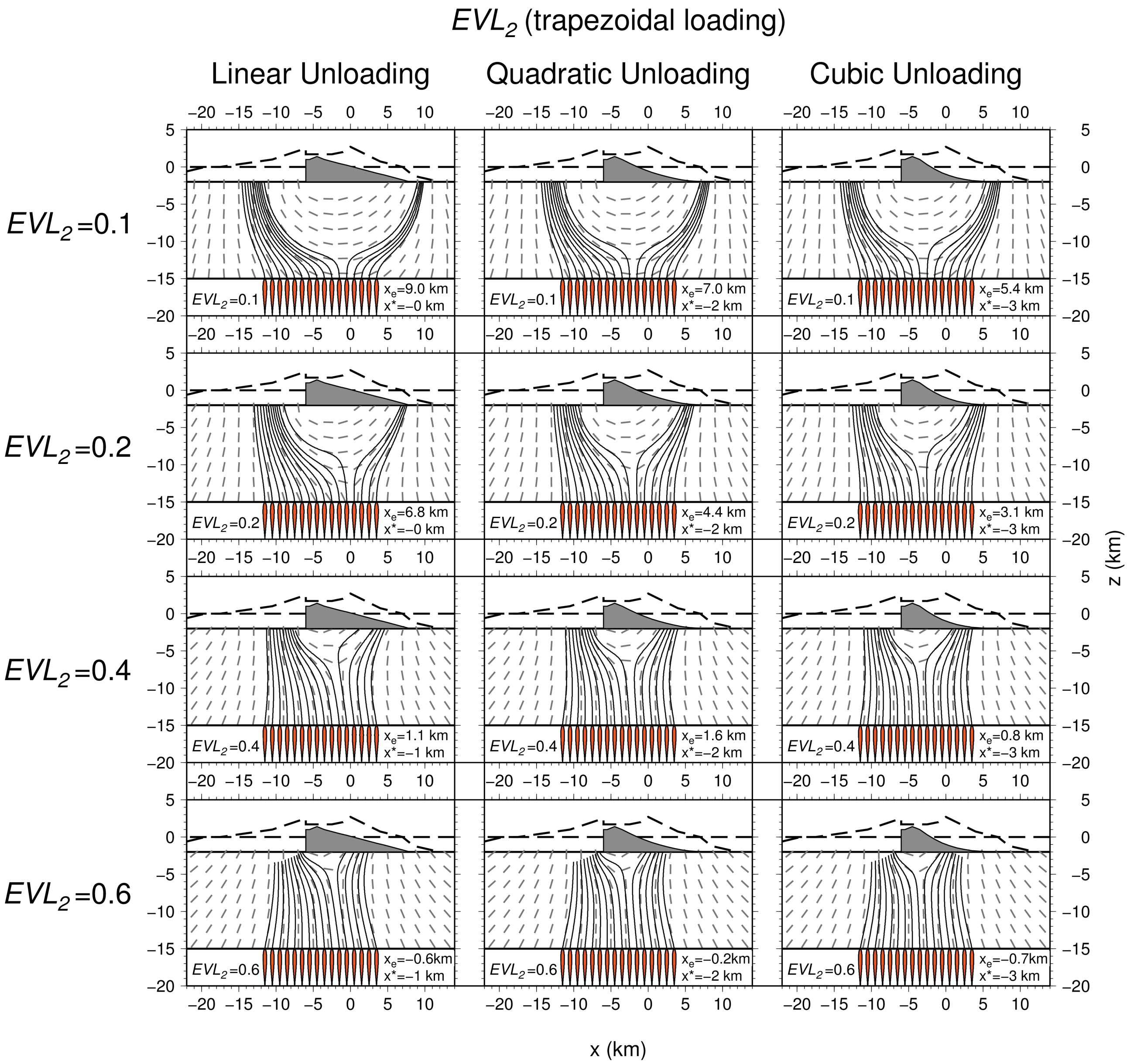


**Supplementary Figure 2:** Model results for $EVL_2$ as function of EVL magnitudes and unloading profiles.

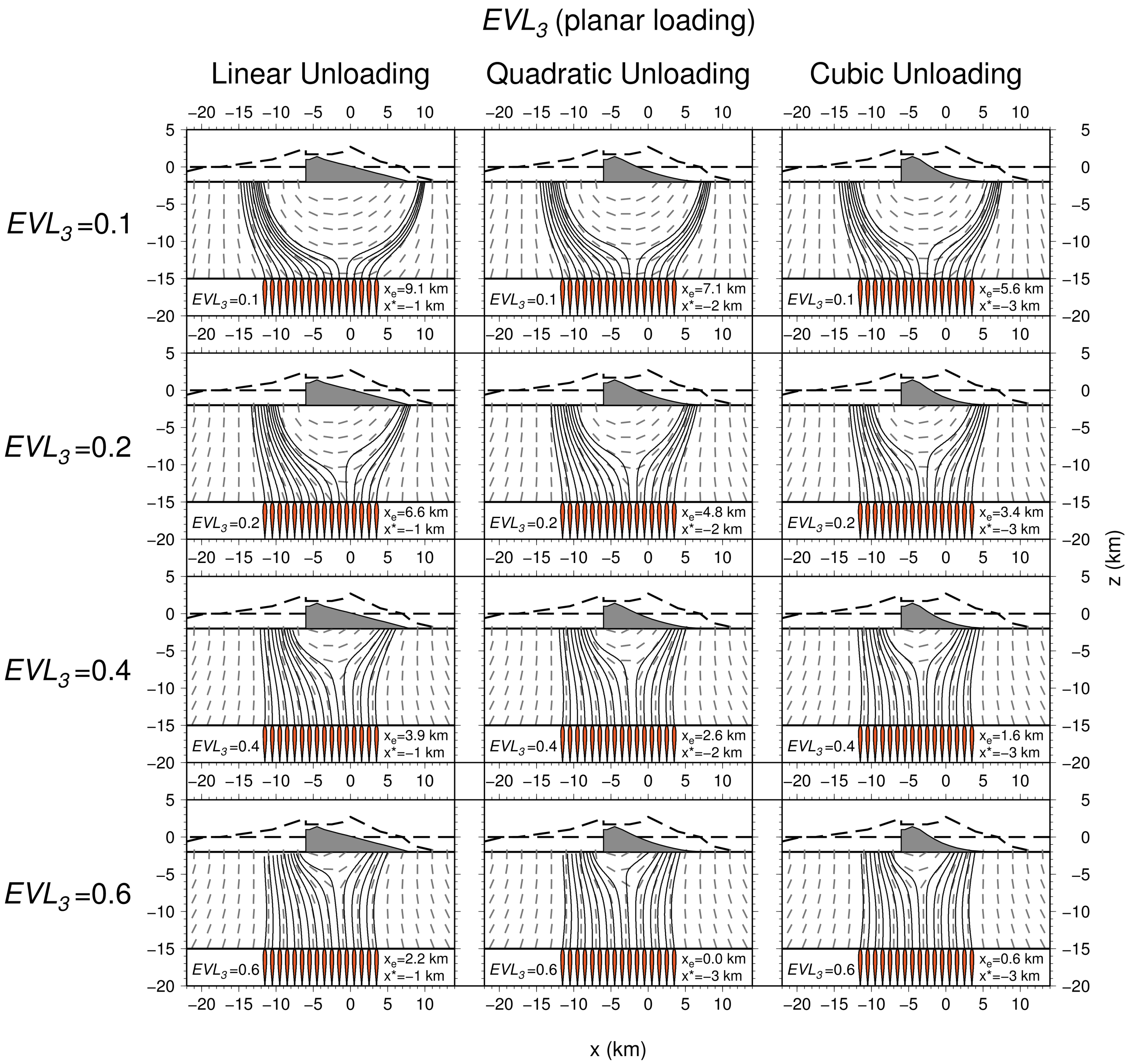


**Supplementary Figure 3:** Model results for $EVL_3$ as function of EVL magnitudes and unloading profiles.

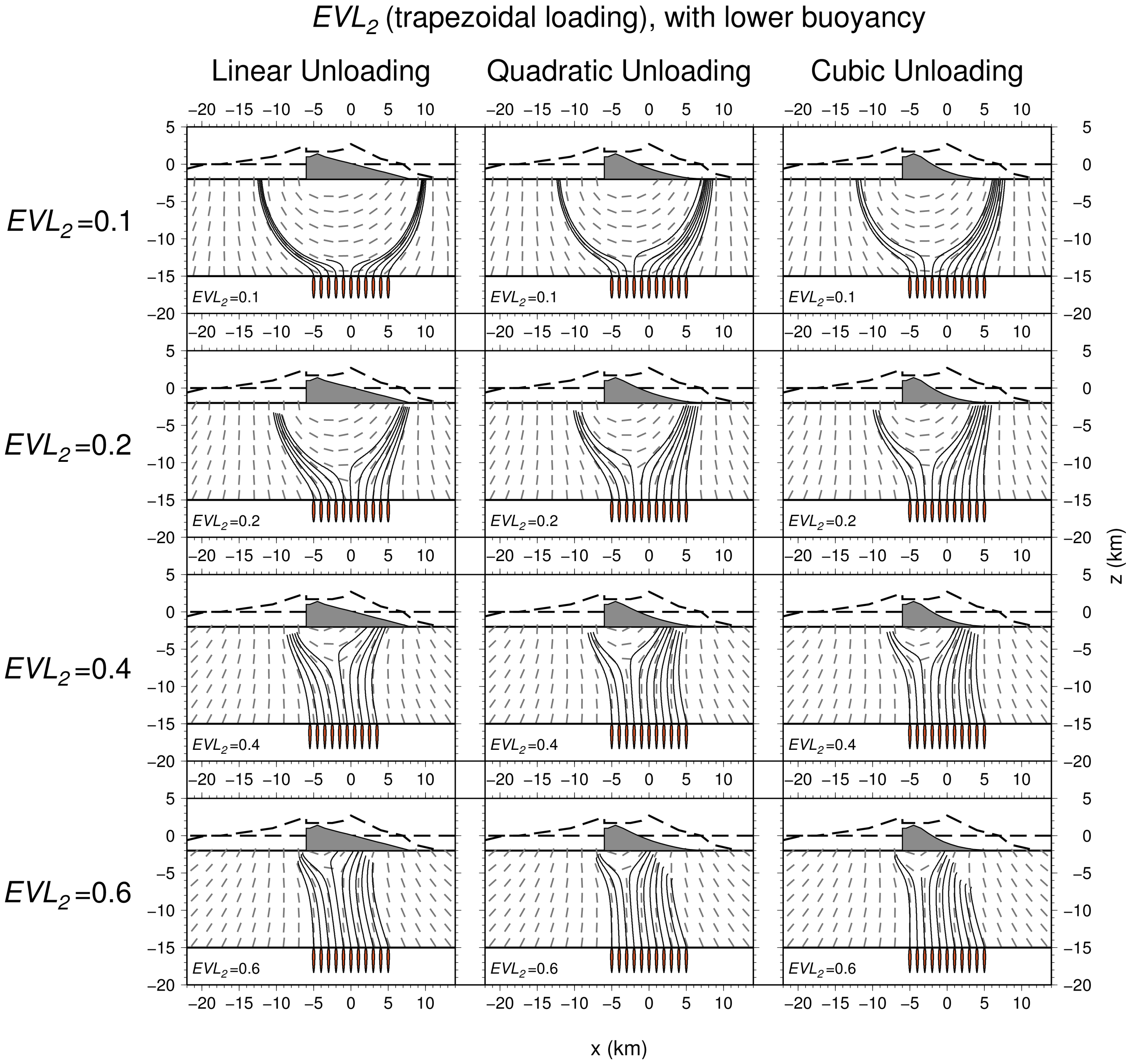


**Supplementary Figure 4:** Model results for $EVL_2$ as function of EVL magnitudes and unloading profiles, with lower buoyancy.

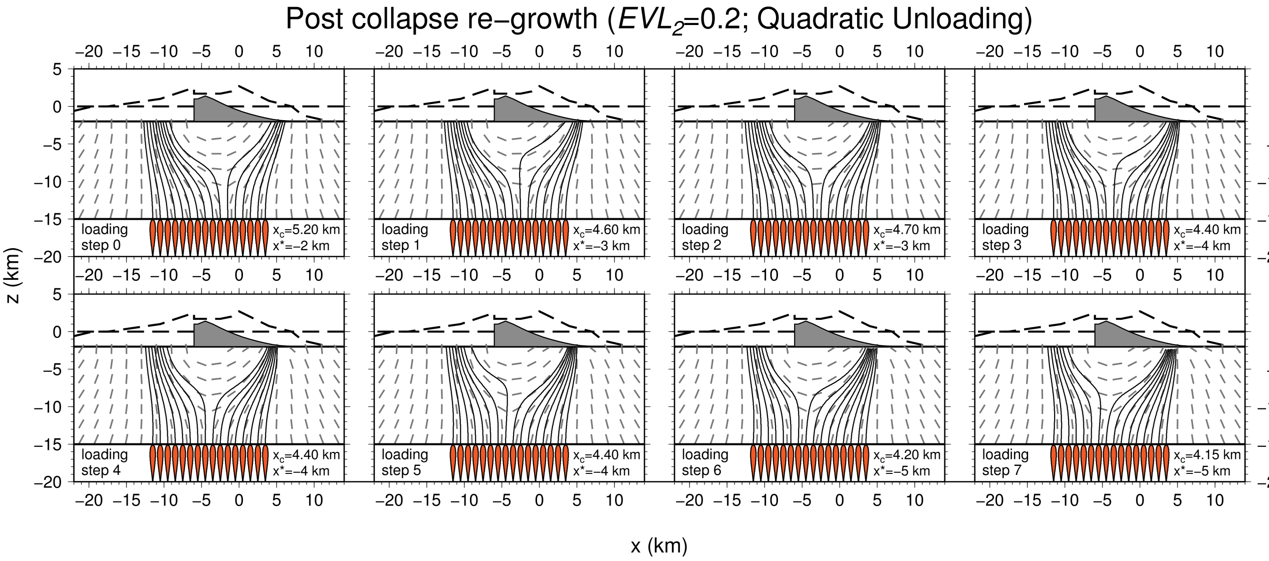


**Supplementary Figure 5:** Model results for the re-growth scenario with $EVL_2$ =0.2 and quadratic unloading profile.

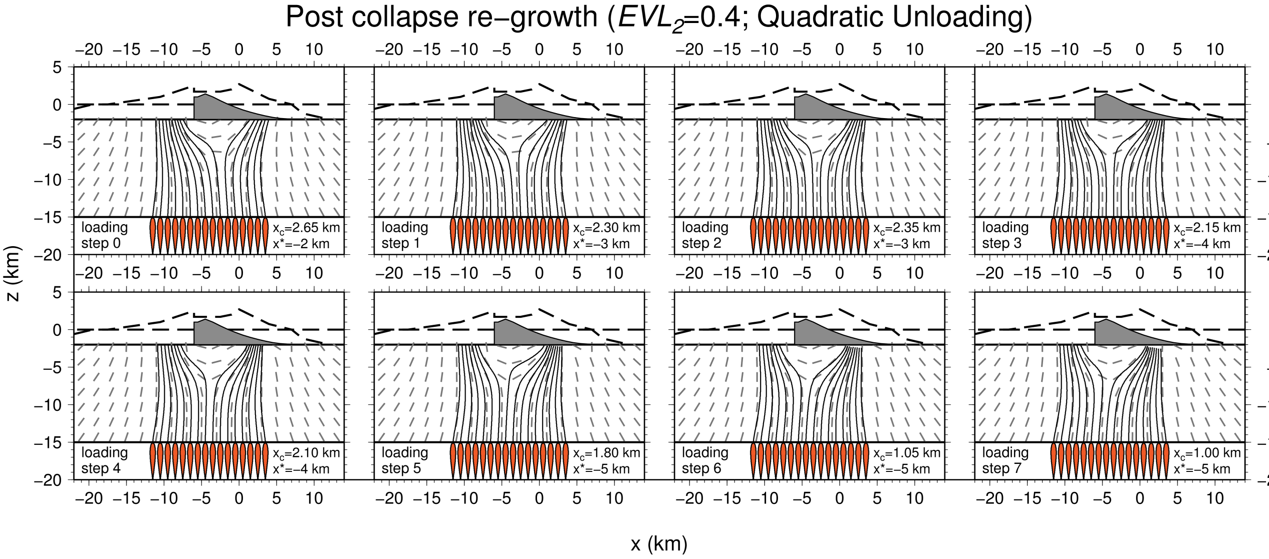


**Supplementary Figure 6:** Model results for the re-growth scenario with $EVL_2$ =0.4 and quadratic unloading profile.
